# Design, Synthesis, Antimycobacterial Evaluation, and In Silico Investigation of Benzisothiazole‐Based Hydrazide Derivatives as Potential Anti‐Tuberculosis Agents

**DOI:** 10.1111/cbdd.70405

**Published:** 2026-09-19

**Authors:** Vivek Sharma, Arun Sharma, Ruchi Poria, Pardeep Kumar

**Affiliations:** ^1^ Department of Chemistry U.G.C. Centre of Advanced Studies Guru Nanak Dev University Amritsar India; ^2^ Swami Dayanand Post Graduate Institute of Pharmaceutical Sciences Rohtak Pandit Bhagwat Dayal Sharma University of Health and Sciences Rohtak Haryana India

**Keywords:** Benzisothiazole, drug resistance, hydrazide, in‐siliico studies, tuberculosis

## Abstract

A series of novel benzisothiazole‐based hydrazide derivatives was rationally designed, synthesized, and evaluated for their antitubercular activity. Among the synthesized compounds, derivative **8k** emerged as the most potent candidate, exhibiting a minimum inhibitory concentration (MIC) of 0.25 μg/mL, along with a high selectivity index (SI) of 42 and confirmed bactericidal activity. To gain preliminary insights into its possible mechanism of action, compound **8k** was subjected to molecular docking and 100 ns molecular dynamics simulations, using enoyl‐acyl carrier protein reductase (InhA) as the proposed molecular target. The computational studies revealed favorable binding interactions and a relatively stable protein–ligand complex, suggesting InhA as a potential molecular target. However, experimental target‐validation studies are required to confirm direct InhA inhibition and establish the proposed mechanism of action. Furthermore, in silico ADMET predictions indicated favorable pharmacokinetic characteristics and drug‐likeness properties for the lead compound. Collectively, these findings highlight the potential of benzisothiazole‐based hydrazide derivatives as promising scaffolds for the development of new antitubercular agents.

## Graphical abstract

A novel series of benzisothiazole‐based hydrazide derivatives was designed, synthesized, and evaluated for antitubercular activity. Compound **8k** emerged as the most potent candidate (MIC = 0.25 μg/mL) with a high selectivity index (SI = 42) and bactericidal activity. Molecular docking and 100 ns simulations identified InhA as the probable target. In silico ADMET analysis indicated favorable drug‐like properties, highlighting these derivatives as promising anti‐tuberculosis agents.

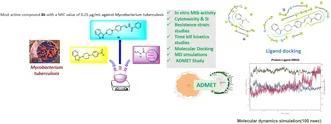

## 1 Introduction

**FIGURE 1:**
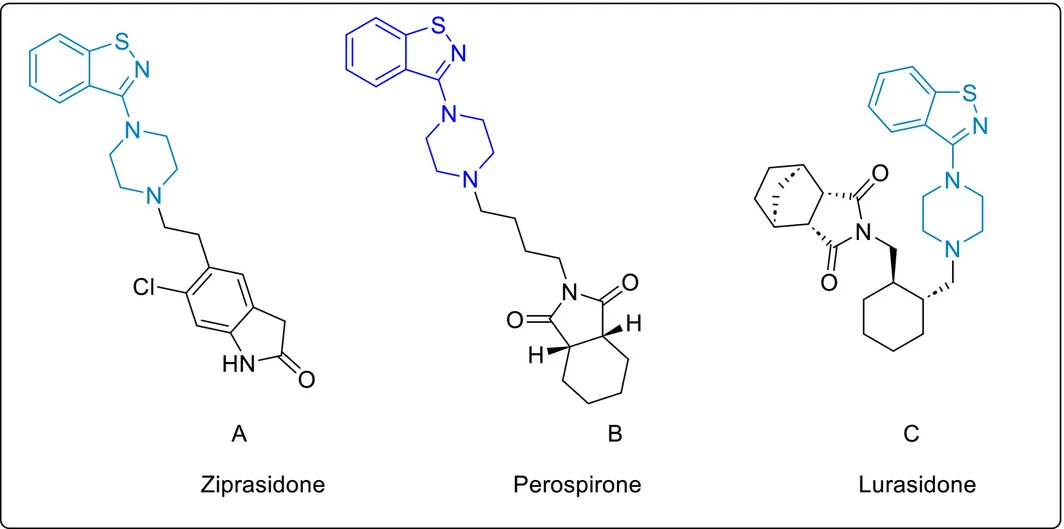
Representative benzisothiazole‐based drugs with diverse pharmacological activities (**A–C**).

Tuberculosis (TB) remains one of the most devastating infectious diseases worldwide, caused by *Mycobacterium tuberculosis* (Mtb), a slow‐growing and persistent pathogen. According to the World Tuberculosis Report 2025, In 2024, an estimated 10.7 million individuals developed tuberculosis, resulting in approximately 1.23 million deaths, with an incidence rate of 131 per 100,000 population and a case fatality rate of 11.5%. In response to the escalating global burden of multidrug‐resistant and extensively drug‐resistant tuberculosis, which underscores an urgent need for new therapeutic agents (World Health Organization 2015; Wei et al. 2024; World Health Organization 2024; Mohajan 2015; Dheda et al. 2024). Current anti‐TB therapy employs a combination of first‐ and second‐line drugs such as isoniazid (InhA inhibitor), rifampicin (RNA polymerase inhibitor), ethambutol (cell wall arabinosyl transferase inhibitor), bedaquiline (ATP synthase inhibitor), and pretomanid (respiratory chain inhibitor via nitric oxide release) (Farhat et al. 2024; Zhang et al. 2024). Although effective, prolonged treatment, adverse effects, and drug–drug interactions—particularly in patients with HIV or diabetes—pose significant clinical challenges (Kumar, Devi, et al. 2026; Dartois and Rubin 2022). The rise of multidrug‐resistant (MDR‐TB) and extensively drug‐resistant (XDR‐TB) strains severely limits treatment efficacy. Isoniazid (pyridine‐4‐carbohydrazide) is a first‐line prodrug activated in the liver by the catalase–peroxidase enzyme (KatG) to form the reactive isonicotinoyl radical, which reacts with nicotinamide adenine dinucleotide (NAD^+^) to generate the INH–NAD adduct (Dartois and Rubin 2022; Kumar, Singampalli, et al. 2026; Den Van Boogaard et al. 2009; Piddock 2006; Raviglione and Smith 2007; Hartkoorn et al. 2014; Kumar, Singampalli, et al. 2025a; Kumar, Singampalli, et al. 2025a; Kumar, Malik, et al. 2025a; Kumar, Singampalli, et al. 2025b; Kumar, Malik, et al. 2025c; Kumar, Malik, et al. 2025b; Kumar, Singampalli, et al. 2025c; Singampalli et al. 2025). This adduct inhibits enoyl‐acyl carrier protein reductase (InhA), a key enzyme in mycolic acid biosynthesis, thereby disrupting cell wall formation and inducing bacterial cell death (García‐Basteiro et al. 2015; Xu et al. 2017; Sankar et al. 2024). Resistance to isoniazid primarily arises from mutations in *katG*, which reduce prodrug activation. Its high hydrophilicity also limits penetration of the lipid‐rich mycobacterial cell wall, necessitating higher doses that may lead to hepatotoxicity, peripheral neuropathy, vasculitis, hematological abnormalities, and clinical hepatitis (Stover et al. 2000; Fujiwara et al. 2018; Cynamon et al. 1999). Given these limitations, InhA remains a validated target for novel inhibitor development. Structure‐guided design, scaffold modification, and hybridization of existing pharmacophores offer promising strategies to generate potent, safer anti‐TB agents capable of overcoming drug resistance (Kumar, Singampalli, et al. 2026; Kumar, Malik, et al. 2025a; Dragostin et al. 2019; Vosátka et al. 2018) Benzisothiazole is a well‐established privileged heterocyclic scaffold that has been extensively explored in medicinal chemistry due to its broad range of biological activities, including antimicrobial potential. Representative bioactive compounds featuring benzisothiazole scaffolds are integral to various therapeutic agents, including antipsychotics (Ziprasidone, Perospirone, and Lurasidone) Figure 1. Several benzisothiazole‐based derivatives have been reported; however, their structural optimization for anti‐tubercular applications remains relatively limited. Similarly, hydrazide and hydrazide‐hydrazone frameworks have received significant attention in tuberculosis drug discovery owing to their favorable hydrogen‐bonding ability, metal‐chelating properties, and capability to interact with essential mycobacterial targets (Devi et al. 2025). Nevertheless, previous studies have mainly focused on either benzisothiazole derivatives lacking hydrazide functionality or hydrazide‐based compounds containing other heterocyclic moieties. A detailed literature investigation revealed that the rational combination of a benzisothiazole nucleus with a hydrazide pharmacophore to generate benzisothiazole–hydrazide hybrid molecules for antimycobacterial evaluation has not been systematically reported. Considering the complementary biological relevance of these two pharmacophores, molecular hybridization was employed in the present study to create a new chemical space of benzisothiazole–hydrazide derivatives. This scaffold design strategy was anticipated to provide additional structural diversity and improve molecular interactions responsible for antimycobacterial activity. The synthesized compounds were comprehensively evaluated for their in vitro antimycobacterial activity, cytotoxicity profile, bactericidal properties, and molecular modelling studies to establish their therapeutic potential. Thus, the present work provides a systematic exploration of benzisothiazole–hydrazide hybrids as a promising anti‐tubercular chemotype and contributes new insights into their structure–activity relationship.

**FIGURE 2:**
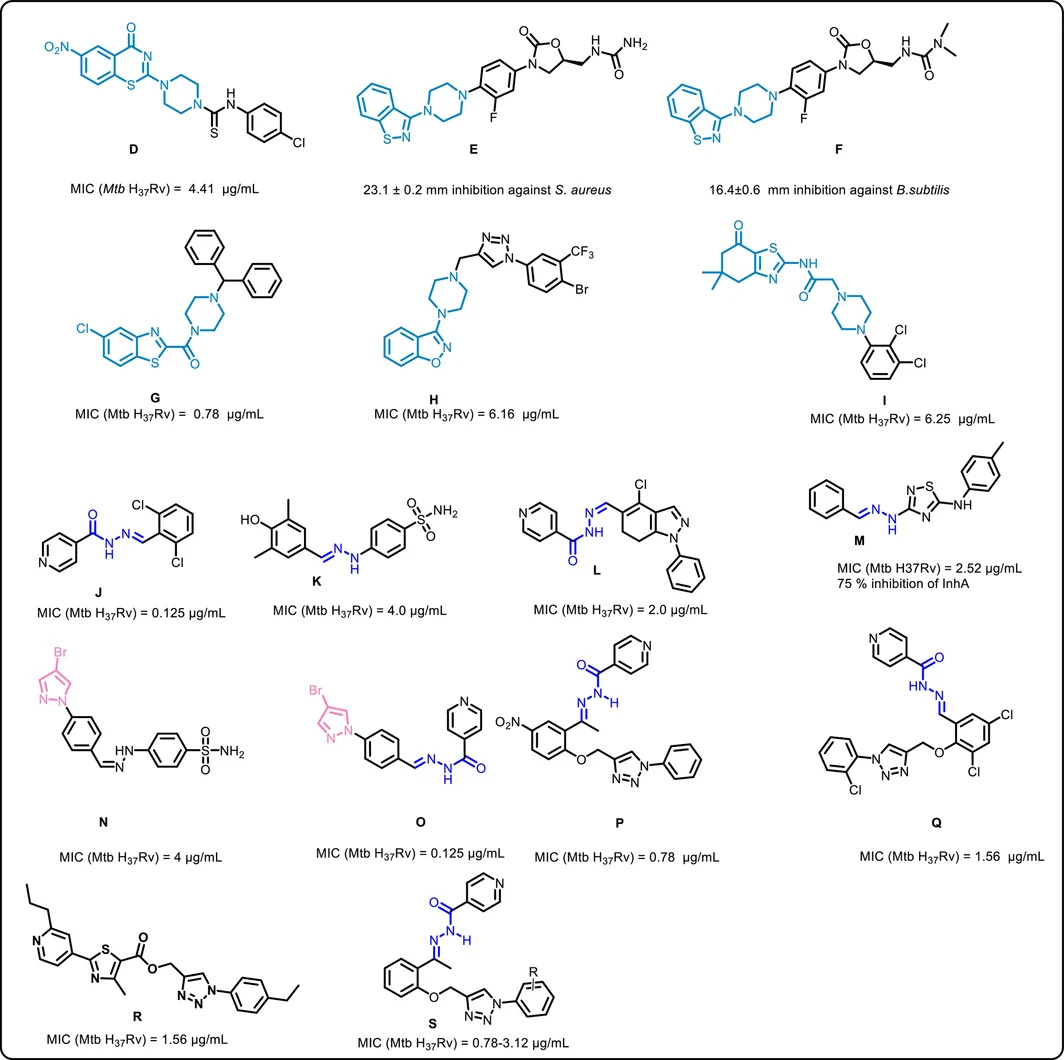
Literature‐reported benzisothiazole and hydrazide‐based derivatives with antitubercular (**D–S**).

Considering the essential role of InhA, an enoyl‐acyl carrier protein reductase involved in the biosynthesis of mycolic acids, the design of benzisothiazole hydrazide derivatives constitutes a rational and strategic approach toward the development of potent antitubercular agents. In this context, Figure 2 Benzisothiazole derivatives have emerged as a promising class of scaffolds for the development of novel antitubercular agents due to their ability to interact with key mycobacterial targets. Chandran et al. (2015) designed and synthesized a series of benzothiazinone–piperazine derivatives that exhibited notable inhibition of *Mycobacterium tuberculosis* DNA gyrase. Within this series, compound **D** emerged as the most active lead, showing an MIC value of 4.41 μM against *M. tuberculosis* . In another study, Patekar et al. (2021) reported oxazolidinone‐linked benzisothiazole–piperazine hybrids with promising antibacterial activity. Among these, compound **E** produced a 23.1 ± 0.2 mm inhibition zone against *Staphylococcus aureus* , while compound **F** exhibited a 16.4 ± 0.6 mm inhibition zone against *Bacillus subtilis*. Furthermore, Pancholia et al. (2016) developed a series of benzothiazol‐piperazine analogues, from which compound **G** demonstrated potent antitubercular activity with an MIC of 0.78 μg/mL against the *M. tuberculosis* H37Rv strain. Likewise, Naidu et al. (2016) synthesized various benzoisoxazole derivatives and identified compound **H** as the most promising molecule, showing an MIC value of 6.16 μM against *M. tuberculosis* . Additionally, Bollikanda et al. (2024) prepared dihydrobenzothiazole‐based *N*‐piperazinylacetamides, among which compound **I** displayed significant inhibitory potential with an MIC of 6.25 μg/mL against *M. tuberculosis* H_37_Rv. Kumar, Malik, et al. (2025a), Kumar, Singampalli, et al. (2025b), and Kumar, Malik, et al. (2025c) have extensively investigated the structure–activity relationship (SAR) of various antitubercular agents in multiple studies. In one report, compound **J** exhibited strong activity against *Mycobacterium tuberculosis* H_37_Rv, with an MIC value of 0.125 μg/mL. In subsequent studies, compounds **K** and **L** were found to possess MIC values of 4.0 μg/mL and 2.0 μg/mL, respectively. Doğan et al. (2020) identified compound **M**, a thiadiazolyl hydrazone, which inhibited InhA by 75% and exhibited a minimum inhibitory concentration (MIC) of 2.52 μM against *Mycobacterium tuberculosis* . Kumar, Singampalli, et al. (2026) reported compound **N** and **O**, a pyrazole‐based pyridine‐4‐carbohydrazide derivative, which exhibited potent antitubercular activity against *Mycobacterium tuberculosis* , with MIC of 0.125 μg/mL and 4.0 μg/mL (Guptha et al. 2014). Patil et al. (2020) synthesized novel isoniazid–triazole derivatives, among which compound **P** showed potent activity against *Mycobacterium tuberculosis* H_37_Rv with an MIC of 0.78 μg/mL. Similarly, Badar et al. (2020) reported the synthesis of isoniazid–1,2,3‐triazole conjugates, with compound Q exhibiting potent antitubercular activity against *Mycobacterium tuberculosis* H_37_Rv (MIC = 1.56 μg/mL). Bakale et al. (2023) were reported a library of 1, 2, 3‐triazole incorporated thiazolylcarboxylate derivatives as potent antitubercular agents among the synthesized compound the most potent compound **R** were reported with MIC of 1.56 μg/mL against *Mycobacterium tuberculosis* . In another report Bakale et al. (2025) were reported a series isoniazid‐triazole derivatives with a MIC value of 0.78–3.12 μg/mL against tuberculosis.

**FIGURE 3:**
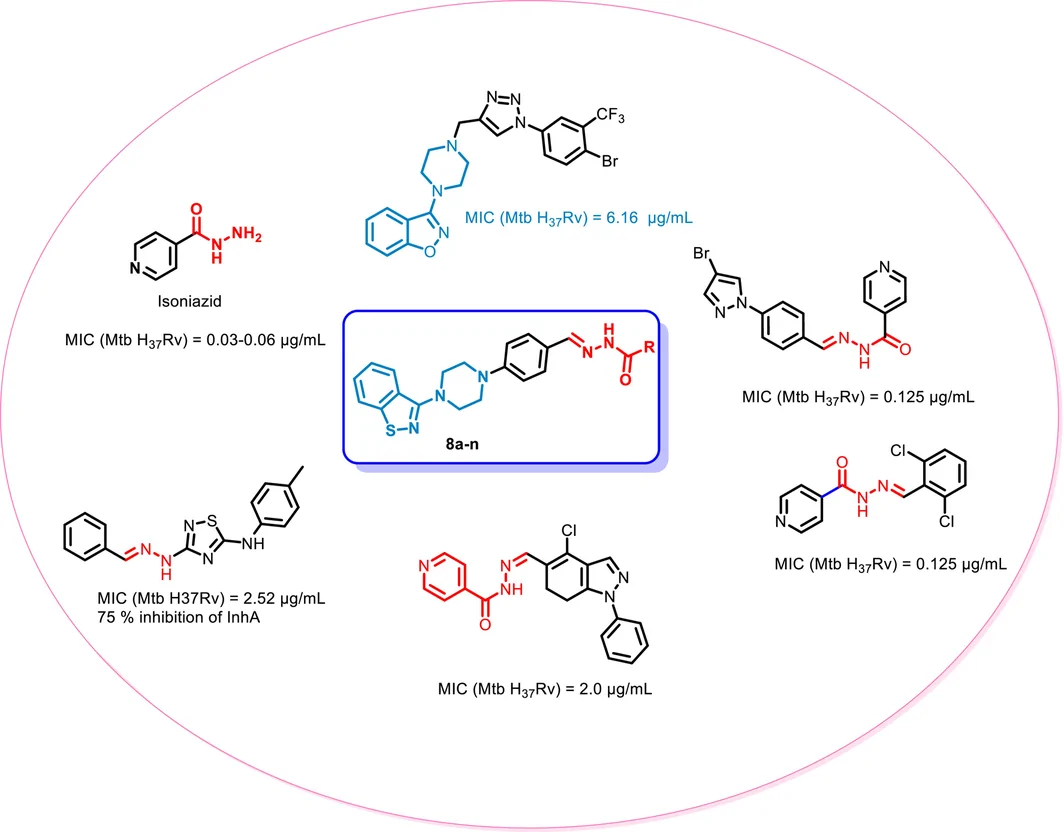
Rationale for the design of the target molecules.

Based on these findings, the present study focuses on the design and synthesis of novel benzisothiazole–hydrazide (Figure 3) with the aim of targeting InhA, the enoyl‐ACP reductase essential for mycolic acid biosynthesis in Mtb. By integrating these scaffolds and hydrazide moieties, these compounds are expected to enhance binding affinity and inhibitory potency against InhA, providing a promising approach for developing new antitubercular agents effective against drug‐resistant strains.

## 2 Results and Discussion

### 2.1 Chemistry

**SCHEME 1:**
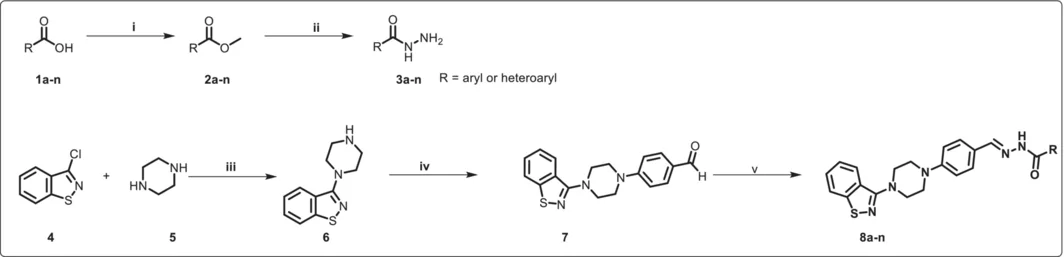
Synthetic route for the preparation of benzisothiazole‐based hydrazide derivatives (**8a–n**): (i) EtOH, Conc. H_2_SO_4_ (reflux 8–10 h 80%–90%); (ii) EtOH, NH_2_NH_2_.H_2_O(2 equiv.), reflux 6 h; (iii)K_2_CO₃ (2 equiv.), dry DMF, 80°C–100°C, 8 h; (iv) 4‐fluoro benzaldehyde (1 equiv.), K_2_CO_3_ (2 equiv.), DMF, 110°C, 15 h, 85%–95%; NH_2_NH_2_.H_2_O (2 equiv.), EtOH, reflux, 4 h, 90%–96%; (v) **3a–n** (1 equiv.), EtOH, 2–3 drops of acetic acid, reflux 8–10 h 60%–90%.

**FIGURE 4:**
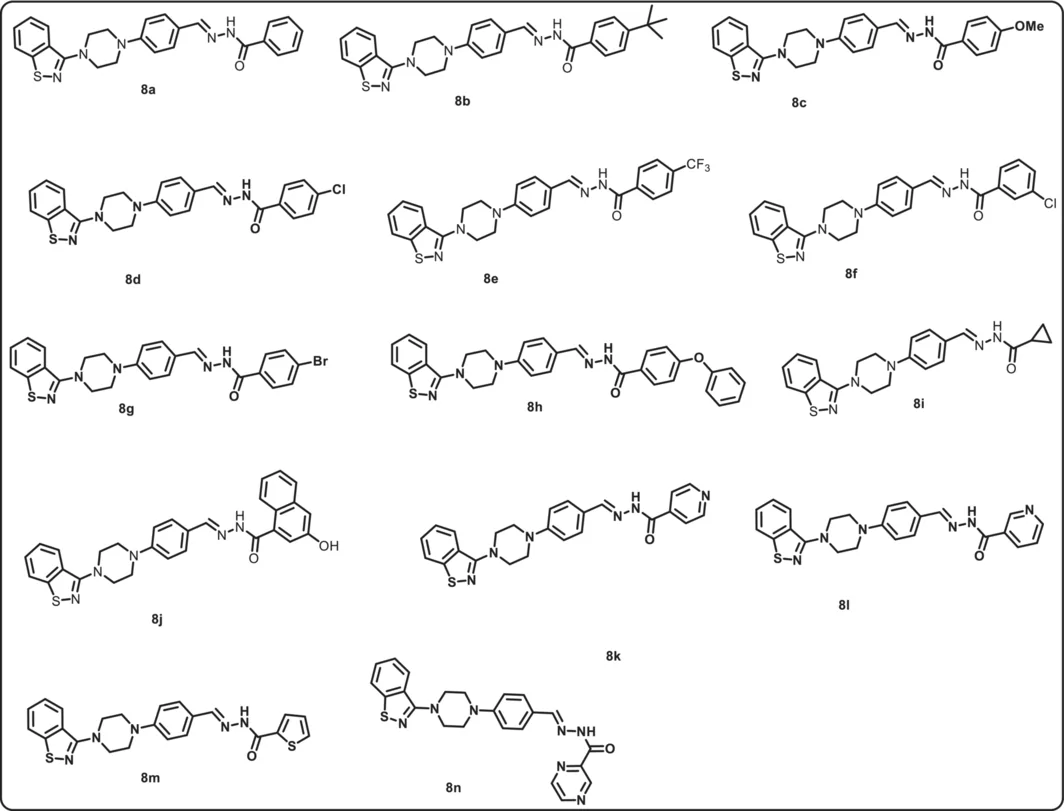
Synthesized molecules in series 1 (**8a–n**).

The synthetic pathway for the target compounds **8a–n** is outlined in Scheme 1. Initially, aryl or heteroaryl carboxylic acids (**1a–n**) were converted into their corresponding methyl esters (**2a–n**) via acid‐catalyzed esterification. These esters were subsequently treated with hydrazine hydrate to yield the corresponding acid hydrazides (**3a–n**). In parallel, 2‐chlorobenzisothiazole (**4**) was reacted with piperazine derivatives (**5**) under basic conditions to afford benzisothiazole–piperazine intermediates (**6**). A nucleophilic substitution between compound **6** and 4‐fluorobenzaldehyde produced the key aldehyde intermediates (**7**). Finally, a condensation reaction between aldehydes **7** and aryl or hetero aryl hydrazides **3a–n** furnished the desired hydrazide‐linked benzisothiazole derivatives (**8a–n**) (Figure 4).

### 2.2 Pharmacology/Biology

#### 2.2.1 In Vitro Antibiotic Susceptibility Against the Mycobacterial Pathogen Panel

The benzisothiazole‐based hydrazide was evaluated for its antimycobacterial potential against the *Mycobacterium tuberculosis* pathogen with Isoniazid as the standard drug. Minimum inhibitory concentration (MIC) was determined as per standard CLSI guidelines. The antimycobacterial screening suggested that compound **8k** exhibited potent inhibition activity against Mtb with an MIC of 0.25 μg/mL against the *Mycobacterium tuberculosis* mc^2^ 6230 as listed in Table 1. In addition to the evaluation against mycobacterial strains, the synthesized benzisothiazole–hydrazide derivatives were further assessed for their antibacterial activity against a representative panel of ESKAPE pathogens, including *Enterococcus faecium* , *Staphylococcus aureus* , *Klebsiella pneumoniae* , *Acinetobacter baumannii* , *Pseudomonas aeruginosa* , and *Enterobacter* spp. Interestingly, none of the synthesized compounds exhibited significant antibacterial activity against the tested ESKAPE pathogens at the evaluated concentrations. This observation suggests that the designed benzisothiazole–hydrazide scaffold displays preferential activity towards mycobacterial species rather than broad‐spectrum antibacterial effects, highlighting its potential selectivity for anti‐tubercular development.

**TABLE 1:** MIC value (μg/mL) of synthesized derivatives against the mycobacterial pathogen panel.

Entry	Compound codes	*M. tuberculosis*
1.	**8a**	> 64
2.	**8b**	> 64
3.	**8c**	> 64
4.	**8d**	> 64
5.	**8e**	> 64
6.	**8f**	> 64
7.	**8g**	> 64
8.	**8h**	> 64
9.	**8i**	> 64
10.	**8j**	64
11.	**8k**	0.25
12.	**8l**	> 64
13.	**8m**	> 64
14.	**8n**	> 64
15.	**Isoniazid**	**< 0.03**

**FIGURE 5:**
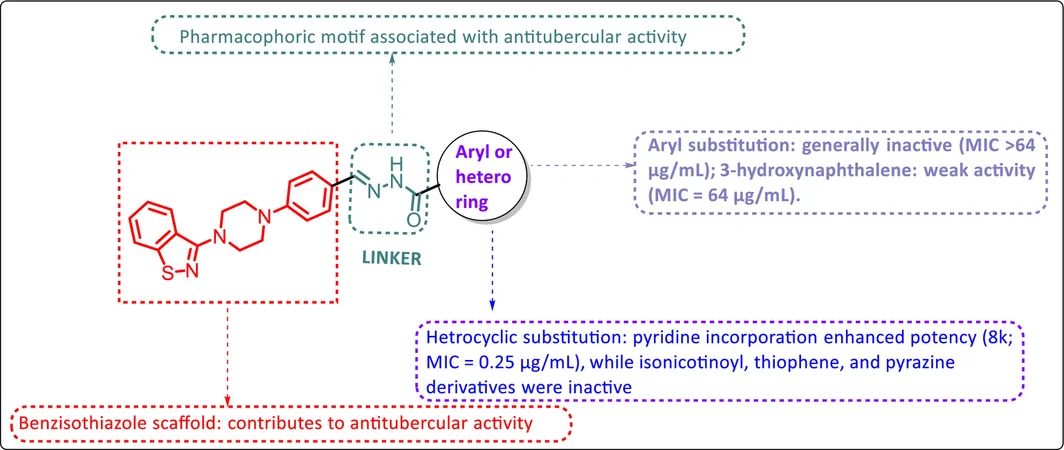
The structure–activity relationships (SAR) of the synthesized compounds.

The structure–activity relationship (SAR) (Figure 5) of the synthesized benzisothiazole–piperazine–hydrazide derivatives (**8a–8n**) revealed that antimycobacterial activity was highly dependent on the nature and physicochemical characteristics of the terminal aromatic/heteroaromatic moiety. The benzisothiazole–piperazine–hydrazide framework was maintained throughout the series, suggesting that the major differences in activity were associated with modifications of the terminal ring.

The unsubstituted phenyl analogue **8a** was inactive (MIC > 64 μg/mL), indicating that a simple phenyl group at the terminal position was insufficient to produce significant antimycobacterial activity. Introduction of different substituents on the phenyl ring did not substantially improve activity. Both electron‐withdrawing substituents, including chloro (**8d** and **8f**), trifluoromethyl (**8e**), and bromo (**8g**), and the electron‐donating methoxy group (**8c**) resulted in inactive compounds (MIC > 64 μg/mL). Thus, the lack of activity across both electron‐withdrawing and electron‐donating substituents suggests that electronic effects alone do not govern the activity of this series. Rather, an appropriate combination of ring polarity, hydrogen‐bonding capacity, molecular shape, and steric properties may be required for productive interaction with the biological target.

Increasing the aromatic surface area also did not result in a proportional improvement in potency. The 3‐hydroxynaphthyl derivative **8j** showed only weak activity (MIC = 64 μg/mL), despite possessing an additional fused aromatic ring and a hydroxyl group capable of hydrogen bonding. This observation suggests that simply increasing hydrophobic/aromatic surface area is not sufficient for enhanced antimycobacterial activity and that steric and/or spatial requirements at the terminal binding region may limit the benefit of a larger bicyclic substituent.

In contrast, replacement of the phenyl‐based terminal group with a pyridine ring in compound **8k** produced a dramatic improvement in antimycobacterial activity (MIC = 0.25 μg/mL), representing a > 256‐fold improvement compared with the inactive phenyl analogue **8a** (MIC > 64 μg/mL). The pronounced activity of **8k** suggests that incorporation of a heteroaromatic nitrogen atom is highly favorable in this chemical series. The pyridine nitrogen may provide an additional hydrogen‐bond acceptor and increase the polarity of the terminal moiety, potentially enabling more favorable interactions with the binding site while maintaining an appropriate aromatic surface and molecular geometry. In comparison, the other heteroaromatic analogues in the series, including isonicotinyl‐, thiophene‐, and pyrazine‐containing derivatives, did not show comparable potency, indicating that the position and number/type of heteroatoms, rather than heteroaromaticity alone, are important for activity. These findings identify **8k** as a promising lead for further structural optimization, particularly through systematic modification of the pyridine ring to investigate the contribution of heteroatom position, polarity, and steric effects to antimycobacterial activity.

### 2.3 Cytotoxicity Against Vero Cells

Among the synthesized benzisothiazole–hydrazide derivatives, compound **8k** was selected for advanced biological evaluation based on its superior antimycobacterial potency, favorable selectivity profile, and promising preliminary structure–activity relationship (SAR) characteristics. Compound **8k** exhibited the most potent activity against *M. tuberculosis* H37Rv with a MIC value of 0.25 μg/mL, along with a favorable selectivity index, making it the most promising candidate for further investigation. Therefore, cytotoxicity assessment, bactericidal evaluation, and additional biological characterization were performed for compound **8k** as a representative lead molecule. Nevertheless, evaluation of additional active analogues would provide further validation of the observed biological trends and will be considered in future optimization studiesThe selectivity index (SI), defined as the ratio of CC_50_ to MIC, was determined to evaluate the safety profile of the most active compound. Cytotoxicity was assessed of compound **8k** using the MTT assay on Vero cells (African green monkey kidney cell line, ATCC CCL‐81). The **CC**_**50**_ represents the concentration at which 50% of the cells exhibit reduced viability. All experiments were performed in triplicate, with Doxorubicin included as positive controls. Detailed results are presented in Table 2.

**TABLE 2:** Cytotoxicity data of the lead molecules (**8k**).

S.No	Code	CC_50_	MIC (μg/mL) against *Mtb* H_37_Rv	Selectivity index
1	**8k**	10.5 μg/mL	0.25 μg/mL	42

### 2.4 Determination of Activity Against SDR‐Mtb Isolates

The present study evaluated the antitubercular activity primarily through MIC determination. Compound **8k** demonstrated promising activity against the tested ethambutol‐resistant *Mycobacterium tuberculosis* isolate. However, a more comprehensive evaluation against a larger panel of multidrug‐resistant (MDR) and extensively drug‐resistant (XDR) *M. tuberculosis* clinical isolates is required to further validate its antitubercular potential. In addition, complementary studies, including minimum bactericidal concentration (MBC) determination and time‐kill kinetic assays, would provide further insights into the bactericidal efficacy and antimicrobial profile of the compound. These investigations are beyond the scope of the present study and will be considered in future work. The identified lead molecule **8k** was found inactive in isoniazid‐resistant strain and exhibited superior activity against the ethambutol‐resistant strain of *M. tuberculosis* . The compound has no effect on multidrug‐resistant strain. The results are summarized in Table 3.

**TABLE 3:** MIC (μg/mL) of the compounds against SDR and MDR Mtb isolates.

S. No.	Compd. code	*M. tuberculosis*	*M.tb* ETB resistance	mc2 8259 (MDR) MIC (μg/mL)
1.	**8k**	0.25	0.25	> 128
**Standard drugs**	**Isoniazid**	0.06	< 0.03	> 128
	**Ethambutol**	2	16	> 128

### 2.5 Time‐Kill Assay

**FIGURE 6:**
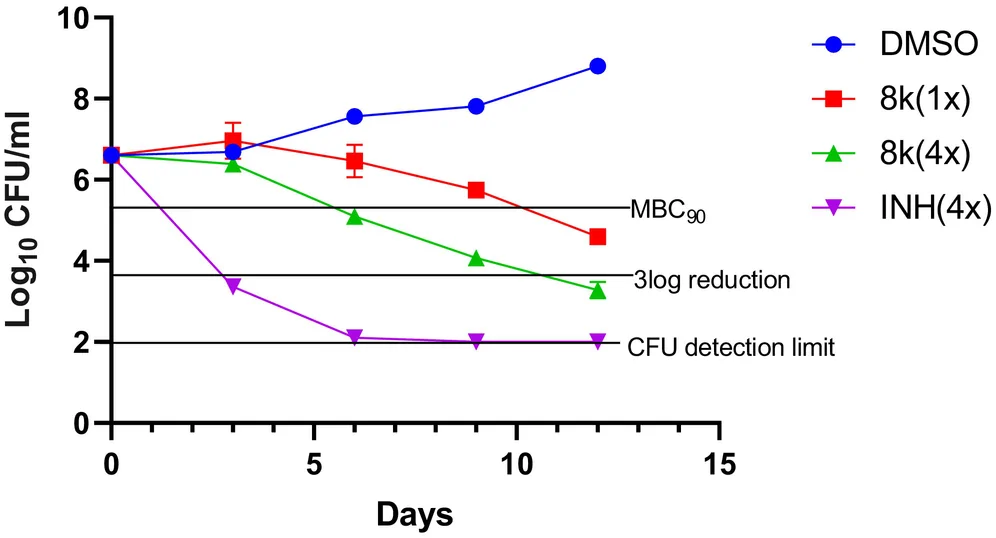
Time kill kinetics of compound **8k**.

To evaluate whether the synthesized compounds exhibit bactericidal or bacteriostatic effects against *Mycobacterium tuberculosis* , time‐kill kinetics assays (Figure 6) were performed for the most active derivatives. Compound **8k** demonstrated bactericidal activity, characterized by a significant and time‐dependent reduction in viable bacterial counts.

## 3 In Silico Studies

### 3.1 Molecular Docking

#### 3.1.1 Validation of the Molecular Docking Protocol

The reliability of the molecular docking protocol was assessed through a redocking validation experiment. The co‐crystallized ligand present in the crystal structure of *Mycobacterium tuberculosis* enoyl‐acyl carrier protein reductase (InhA; PDB ID: 2X23; resolution 1.81 Å) was extracted from the active site and subsequently redocked into the same binding pocket using the Glide XP docking protocol and receptor grid parameters employed for the synthesized compounds. The predicted redocked pose was superimposed onto the experimentally determined crystallographic pose, and the root‐mean‐square deviation (RMSD) between the two poses was calculated. The redocking procedure yielded an RMSD of **0.96 Å**, indicating close agreement between the predicted and experimentally observed ligand binding orientations. As an RMSD value below 2.0 Å is generally considered indicative of successful reproduction of the experimentally observed binding pose, the obtained RMSD supports the reliability of the applied docking protocol for investigating the binding modes of the synthesized compounds within the InhA active site.

Molecular docking, MM/GBSA binding energy calculations, and molecular dynamics simulations were performed using the Schrödinger molecular modeling suite 2022‐1 (Schrödinger LLC, New York, NY, USA). The three‐dimensional structures of the test compounds (**8k** and isoniazid) were prepared using the LigPrep module, where possible ionization and tautomeric states were generated using Epik at pH 7.4, followed by energy minimization using the OPLS4 force field. The protein structure was prepared using the Protein Preparation Wizard by assigning bond orders, adding missing residues and loops, optimizing hydrogen bonding networks, generating appropriate protonation states at pH 7.4, and performing restrained minimization using the OPLS4 force field. Water molecules beyond 5 Å from the active site were removed, and the prepared protein structure was used for receptor grid generation. The receptor grid was generated around the co‐crystallized ligand binding site using the Glide module with grid center coordinates of X = −19.640, Y = −4.133, and Z = −22.575, and a grid box dimension of 30 × 30 × 30 Å. Extra precision (XP) docking was subsequently performed using Glide. The binding free energies of the docked complexes were calculated using the Prime MM/GBSA approach according to the equation: **ΔG_bind = ΔE_MM + ΔG_solv + ΔG_SA** where ΔE_MM represents the molecular mechanics energy difference, ΔG_solv represents the solvation energy contribution, and ΔG_SA represents the solvent‐accessible surface area energy contribution during complex formation (Bowers et al. 2006; Kumar and Sobhia 2016; Aijijiyah et al. 2024; Halder and Elma 2021; Mardianingrum et al. 2021).

#### 3.1.2 Ligand Preparation

The 2D structure of isoniazid, and the most active compounds (**8k**) were prepared and saved in .mol 3000 format using ChemDraw. These ligands were then prepared using the ligand preparation wizard of Schrödinger molecular modelling suite 2022‐1, followed by generation of low‐energy ionization states and tautomer states using Epik at pH 7.4. The ligands were further stabilized by minimizing the energy state using the OPLS4 force field of the macromodel module, which was then further used during the docking study.

#### 3.1.3 Protein Preparation

The three‐dimensional X‐ray crystal structures of *Mycobacterium tuberculosis* InhA (PDB IDs: **2X23**) were retrieved from the RCSB Protein Data Bank with resolutions of 1.81 Å (2X23) and 1.64 Å, respectively. Each structure was prepared using the Protein Preparation Wizard in Maestro. Missing side chains and loops were modeled using Prime, and crystallographic water molecules beyond 5 Å from any ligand or active site residue were removed. Protonation states and tautomers were assigned for pH 7.4 using Epik, and hydrogen atoms were optimized to correctly orient side chains and hydrogen bonding networks. The structures were then subjected to restrained energy minimization under the OPLS4 force field to relieve steric clashes and stabilize geometry while preserving experimentally resolved features.

For each prepared structure, receptor grids were generated using the Receptor Grid Generation panel in the Glide module (Schrödinger Suite 2022‐1). The grid boxes were centered around the active site residues, with coordinates such as X = −19.65, Y = −4.13, Z = −22.57; 57 were generated around the active site of the protein.

#### 3.1.4 Ligand Docking and Binding Energy Calculation

Molecular docking was performed to predict the initial binding conformations and interactions of the synthesized derivatives within the active site of InhA, a critical enzyme involved in mycobacterial fatty acid biosynthesis. The crystallographic structures of InhA (PDB IDs: **2X23**) were selected to represent different protein conformations and ligand‐bound states.

Docking was carried out using the extra precision (XP) protocol implemented in Glide (Maestro 13.1) to generate plausible binding poses. To further evaluate and rank the docked complexes, binding free energies (ΔG_bind) were estimated using the MM‐GBSA method available in the Prime module (Schrödinger Release 2022‐1).

**FIGURE 7:**
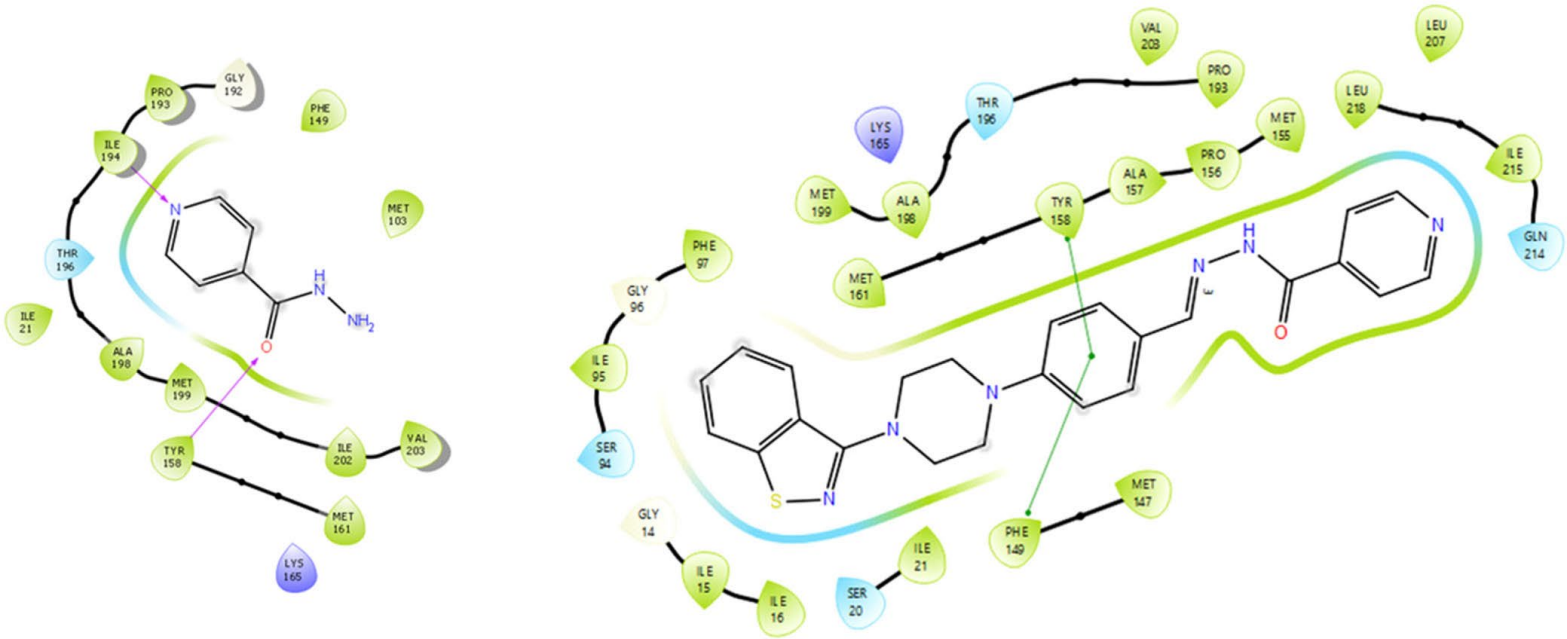
2D interaction diagram of docking lead molecule **8k(PDB ID: 2X23)** and isoniazid.

Molecular docking analysis of the most active compound **8k** was performed against InhA (PDB ID: 2X23), and isoniazid (INH) was used as a reference compound. Compound **8k** showed a docking score of −7.781 kcal/mol, which was more favourable than that of INH (−3.09 kcal/mol). The predicted binding mode of **8k** was stabilized by hydrogen‐bonding and aromatic interactions with residues located within the InhA binding pocket. In particular, Tyr158 was involved in hydrogen‐bonding interactions, while Phe149 contributed through π–π interactions with the aromatic moiety of **8k** (Figure 7). In contrast, the interaction of INH with the binding pocket involved Tyr158 and hydrophobic contacts with Ile194. These interactions were consistent with the chemical nature of the respective amino acid side chains and the ligand–residue geometry observed in the docking poses.

The MMGBSA analysis further supported the predicted binding of **8k**, as shown in Table 4 which exhibited a binding free energy (ΔGbind) of −38.69 kcal/mol compared with −24.66 kcal/mol for INH. The more favourable MMGBSA value for **8k** suggests stronger predicted ligand–InhA binding; however, these computational values are interpreted as supportive evidence of binding interactions rather than direct experimental measures of binding affinity.

**TABLE 4:** Docking score (d.s.), MMGBSA binding free energy (∆Gbind), and binding site residues (BSR) of test compounds with target enzyme InhA.

S.No	Comp. code	Dock. score	PDB ID	∆Gbind (kcal/mol)	Key interacting residues	Interaction
1	**8k**	−7.781	2X23	−38.69	TYR158, PHE 149	H‐bond, π–π
2	**INH**	−3.09	2X23	−24.66	TYR 158, ILE 194	H‐bond, hydrophobic

### 3.2 Molecular Dynamics Simulations

To complement the initial docking predictions and to capture the dynamic nature of ligand–protein interactions, molecular dynamics simulations were subsequently performed. These simulations provided detailed insights into the stability and evolution of binding interactions within the InhA active site, thereby enriching the understanding gained from docking studies.

Molecular dynamics (MD) simulations were performed using the Desmond module integrated within the Schrödinger Suite 2022‐1. All protein–ligand complexes were prepared and solvated using the OPLS4 force field. Each system was neutralized by the addition of two sodium ions (Na^+^) and supplemented with 0.15 M NaCl to mimic physiological monovalent ion concentrations. Following energy minimization, systems were subjected to initial heating using Brownian dynamics in the NVT ensemble for 60 ps at 310 K, with a harmonic positional restraint of 5 kcal·mol^−1^·Å^−2^ applied to the heavy atoms. This was followed by equilibration in the NPT ensemble for 1 ns at 300 K and 1.013 bar, during which the restraints were gradually reduced to zero. Production runs were then carried out for 100 ns under NPT conditions using a Langevin thermostat and barostat with a collision frequency of 1 ps^−1^ to maintain constant temperature and pressure.

**FIGURE 8:**
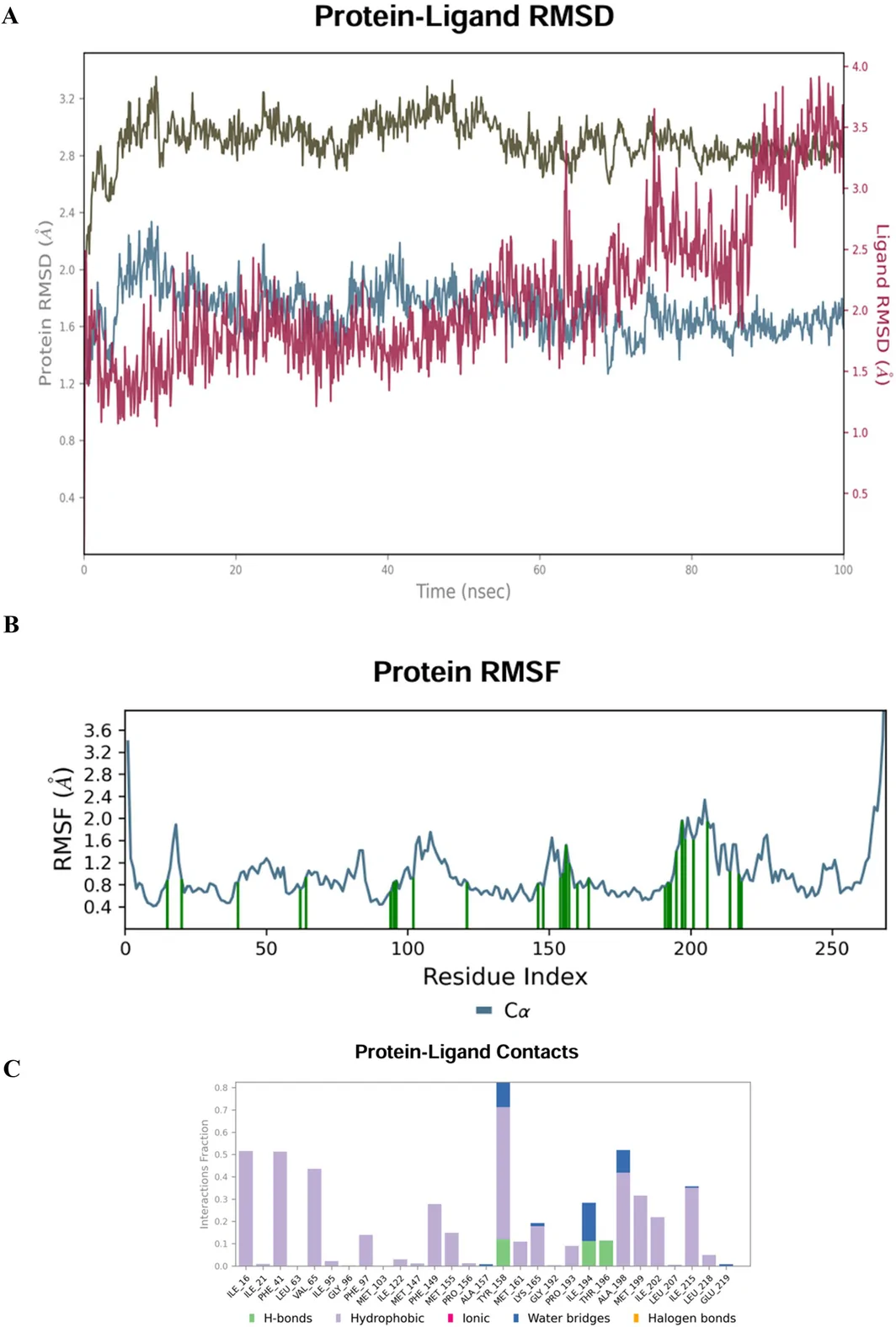
Trajectory plots of **2X23‐8k** complex over 100 nsec molecular dynamics simulation representing protein‐ligand RMSD (A), protein RMSF (B) and protein‐ligand contact histogram (C).

Compound **8k** was simulated (Figure 8) in complex with the enoyl‐acyl carrier protein reductase (**InhA, PDB ID: 2X23**) using the described protocol. The **2X23–8k** complex demonstrated stable binding over the 100 ns simulation, maintaining key hydrogen bond interactions with residues TYR158, ILE194, as illustrated in Figure 8A–C. Collectively, these results strongly indicate that InhA most probably serves as the primary molecular target of the synthesized compounds, thereby substantiating their potential as effective antitubercular agents.

The molecular docking and molecular dynamics results provide preliminary structural evidence for a plausible interaction of compound **8k** with the InhA binding site; however, these computational findings do not establish direct InhA inhibition, and experimental target‐validation studies are required to determine whether InhA is a relevant molecular target of **8k**.

### 3.3 Pharmacokinetic Properties Prediction

Pharmacokinetic parameters like absorption, distribution, metabolism, excretion, and organ toxicity, along with LD_50_ , were predicted using the QikProp module of Maestro 13.1 (Schrödinger Suite 2022‐1) and ProTox 3.0, respectively.

### 3.4 Drug Likeness of Synthesized Derivatives

In silico evaluation of the physicochemical and drug‐likeness properties of the lead compound **8k** was performed using the QikProp module of the Schrödinger molecular modelling suite. The evaluated parameters included molecular weight, lipophilicity (octanol/water partition coefficient), hydrogen‐bond donor and acceptor counts, number of rotatable bonds, aqueous solubility (ESOL), and topological polar surface area (TPSA). Isoniazid (INH) was included as a reference drug for comparison. The predicted physicochemical and drug‐likeness parameters are summarized in (Table 5).

**TABLE 5:** Predicted physicochemical and drug‐likeness properties of compound **8k**, with isoniazid (INH) included as a reference drug.

Parameters	Recommended range	8k	Isoniazid (INH)
Rule of five	Maximum is 4	No violation	No violation
Molecular weight	≤ 500	442.54 g/mol	137.14 g/mol
Donor HB	0.0–6.0	1	2
Acceptor HB	2.0–20.0	4	3
No. of rotatable bonds	≤ 10	6	2
Lipophilicity Consensus Log P_o/w_	≤ 5	3.36	−0.44
Water solubility LogS (ESOL)	—	−5.34	−0.56
TPSA (Å^2^)	≤ 140	101.96	68.01

Compound **8k** showed acceptable physicochemical characteristics, with a molecular weight of 442.54 g/mol, one hydrogen‐bond donor, four hydrogen‐bond acceptors, six rotatable bonds, a predicted logP of 3.36, and a TPSA of 101.96 Å^2^. The compound showed no violation of Lipinski's Rule of Five, supporting its favorable drug‐like profile. However, these in silico predictions are preliminary and should be considered hypothesis‐generating rather than definitive evidence of favorable pharmacokinetic behavior. Experimental in vitro and in vivo studies will be required to validate these predictions.

In addition, the predicted toxicity profile of compound **8k**, obtained using ProTox‐3.0, is presented in (Table 6). The predicted hepatotoxicity, neurotoxicity, and cytotoxicity endpoints were classified as inactive, with probabilities of 0.65, 0.67, and 0.62, respectively. These predictions provide preliminary information regarding the potential toxicity profile of **8k** but require experimental validation (Ursu et al. 2011; Lipinski 2004; Muresan et al. 2012; Clark and Pickett 2000).

**TABLE 6:** The predicted toxicity profile of compound **8k**.

Category	Endpoint	Prediction	Probability
Organ toxicity	Hepatotoxicity	Inactive	0.65
	Neurotoxicity	Inactive	0.67
	Cytotoxicity	Inactive	0.62

## 4 Conclusion

This study successfully designed, synthesized, and evaluated a series of benzisothiazole–hydrazide derivatives as potential anti‐tubercular agents. Among the synthesized analogues, compound **8k** emerged as the most promising candidate, exhibiting potent antimycobacterial activity (MIC = 0.25 μg/mL), a favorable selectivity profile, and activity against drug‐resistant strains of *Mycobacterium tuberculosis* . The structure–activity relationship (SAR) analysis revealed that simple aromatic substitutions were insufficient to confer significant activity, whereas specific structural features present in **8k** contributed to enhanced potency, highlighting the importance of precise structural modification at the terminal position. Molecular docking and long‐timescale molecular dynamics simulations suggested that **8k** may interact favorably and stably within the InhA active site, supporting InhA as a potential molecular target. Furthermore, in silico ADME predictions indicated favorable drug‐like properties for the lead compound, supporting its further investigation. Despite these promising findings, certain limitations should be acknowledged. The proposed mechanism of action is based on computational studies and lacks direct experimental validation. Additionally, the pharmacokinetic behavior, toxicity profile, and in vivo efficacy of the lead compound remain to be established. Future studies will therefore focus on experimental target validation, detailed pharmacokinetic evaluation, and in vivo efficacy studies, along with further structural optimization to enhance potency and selectivity. Overall, the present work provides a useful framework for the development of benzisothiazole‐based chemotypes as potential anti‐tubercular agents. These findings support further investigation of rationally designed benzisothiazole derivatives as promising scaffolds for the development of new agents against drug‐resistant tuberculosis.

## 5 Experimental

### 5.1 Chemistry

#### 5.1.1 General

All chemicals, reagents, and solvents were purchased from commercial suppliers and used without further purification. Reactions were monitored by TLC using Merck precoated silica gel 60 F254 aluminium plates (0.5 mm) and visualized under UV light. ^1^H and ^13^C NMR spectra were recorded on a Bruker Avance 500 MHz spectrometer using dimethyl sulfoxide (DMSO‐*d*_6_) as solvent and tetramethylsilane (TMS) as internal standard. Chemical shifts (*δ*) are reported in parts per million (ppm) and referenced to TMS (δ = 0.00 ppm for ^1^H and ^13^C NMR). Spin multiplicities are reported as singlet (s), doublet (d), doublet of doublets (dd), triplet (t), quartet (q), and multiplet (m). Coupling constants (*J*) are given in hertz (Hz). High‐resolution mass spectra (HRMS) were recorded on an Agilent 6540 quadrupole time‐of‐flight (Q‐TOF) mass spectrometer using electrospray ionisation (ESI). Column chromatography was performed on silica gel (60–120 mesh). Melting points were measured on a Stuart SMP30 apparatus. Tha data that supports the research are described in Appendix S1.

#### 5.1.2 General Procedure for the Synthesis of Target Molecules (8a–n)

##### 5.1.2.1 General Procedure for the Synthesis of Methyl Esters (2a–n)

A mixture of the appropriate aryl or heteroaryl carboxylic acid (**1a–n**, 1 mmol) and methanol (10 mL) was refluxed in the presence of concentrated sulfuric acid (2–3 drops) for 8–10 h. The mixture was cooled, poured into ice‐cold water, and neutralized. The resulting solid was filtered, washed with water, and dried to afford methyl esters (2a–p) in 80%–90% yield.

##### 5.1.2.2 General Procedure for the Synthesis of Acid Hydrazides (3a–n)

To a stirred solution of ester (**2a–n**, 1 mmol) in ethanol (10 mL), hydrazine hydrate (2–3 mmol) and a few drops of water were added. The reaction mixture was refluxed for 6 h. After cooling, the solid was filtered, washed with ethanol, and dried to obtain hydrazides (**3a–n**).

##### 5.1.2.3 General Procedure for the Synthesis of Benzisothiazole–Piperazine Intermediate (6)

2‐Chlorobenzisothiazole (**4**, 1 mmol) and the piperazine (**5**, 1.1 mmol) were dissolved in dry DMF (10 mL). Potassium carbonate (2 mmol) was added, and the reaction mixture was refluxed at 80°C–100°C for 8 h. After cooling, the mixture was poured into cold water. The precipitate was filtered, washed with water, and dried to yield intermediate (**6**) in 90%–96% yield.

##### 5.1.2.4 General Procedure for the Synthesis of Aldehyde Intermediate (7)

Compound **6** (1 mmol) was reacted with 4‐fluorobenzaldehyde (1.1 mmol) in DMF (10 mL) in the presence of K_2_CO_3_ (2 mmol). The reaction mixture was stirred at 110°C for 15 h. After cooling, the mixture was poured into water. The resulting solid was filtered and dried to afford aldehyde (**7**) in 85%–95% yield.

##### 5.1.2.5 General Procedure for the Synthesis of Final Compounds in Series 1 (8a–n)

A mixture of aldehyde **7** (1 mmol) and the appropriate hydrazide (**3a–n**, 1.1 mmol) was refluxed in ethanol (10 mL) in the presence of 2–3 drops of acetic acid for 8–10 h. After cooling, the solid was filtered, washed with ethanol, and dried to obtain the final compounds (**8a–n**) in 80%–90% yield.

The structures of the synthesized compounds were confirmed through comprehensive spectroscopic characterization, including ^1^H NMR, ^13^C NMR, and HRMS analyses.

We acknowledge that definitive confirmation of the solid‐state conformation by techniques such as single‐crystal X‐ray diffraction was not performed in the present study, as it was beyond the scope of this work. However, the proposed structures were assigned based on the obtained spectroscopic data, and their biologically relevant conformations were further investigated through molecular docking studies.

(*E*)‐N′‐(4‐(4‐(benzo[d]isothiazol‐3‐yl)piperazin‐1‐yl)benzylidene)benzohydrazide (**8a**).


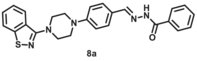
Brown solid; yield 78%; mp: 196°C–198°C; ^1^H NMR (500 MHz, DMSO‐*d*_6_) *δ* 11.60 (s, 1H), 8.35 (s, 1H), 8.14 (d, *J* = 8.2 Hz, 1H), 8.09 (d, *J* = 8.1 Hz, 1H), 7.95 (d, *J* = 8.8 Hz, 2H), 7.61 (d, *J* = 2.3 Hz, 2H), 7.58 (d, *J* = 7.3 Hz, 1H), 7.47 (d, *J* = 7.6 Hz, 2H), 7.24 (d, *J* = 7.4 Hz, 1H), 7.10 (s, 3H), 3.61 (d, *J* = 5.1 Hz, 4H), 3.51 (s, 4H); ^13^C NMR (125 MHz, DMSO‐*d*_6_) *δ* 163.82, 163.29, 152.57, 148.59, 134.22, 131.99, 131.51, 128.89, 128.85, 128.43, 128.00, 124.97, 124.60, 121.59, 115.23, 49.87, 47.64; HRMS‐QTOF (ESI): *m/z* calcd. for [M + H]^+^ C_25_H_24_N_5_OS 442.1696; observed 442.1697.

(*E*)‐N′‐(4‐(4‐(benzo[d]isothiazol‐3‐yl)piperazin‐1‐yl)benzylidene)‐4‐(tert‐butyl)benzohydrazide (**8b**).


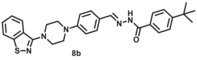
White solid; yield 71%; mp: 181°C–183°C; ^1^H NMR (500 MHz, DMSO‐*d*_6_) *δ* 11.57 (s, 1H), 8.35 (s, 1H), 8.14 (d, *J* = 8.2 Hz, 1H), 8.09 (d, *J* = 8.1 Hz, 1H), 7.84 (d, *J* = 8.5 Hz, 2H), 7.62 (d, *J* = 9.0 Hz, 2H), 7.60–7.57 (m, 1H), 7.53 (d, *J* = 8.4 Hz, 2H), 7.48 (t, *J* = 7.2 Hz, 1H), 7.09 (d, *J* = 8.7 Hz, 2H), 3.64–3.59 (m, 4H), 3.51 (d, *J* = 4.8 Hz, 4H), 1.32 (s, 9H); ^13^C NMR (125 MHz, DMSO‐*d*_6_) *δ* 163.81, 162.30, 158.08, 154.28, 148.95, 133.26, 131.93, 130.12, 128.91, 128.44, 127.79, 125.74, 124.96, 124.83, 124.60.23, 121.59, 115.20, 49.86, 47.61, 34.97, 31.17; HRMS‐QTOF (ESI): *m/z* calcd. for [M + H]^+^ C_29_H_32_N_5_OS 498.2322; observed 498.2288.

(*E*)‐N′‐(4‐(4‐(benzo[d]isothiazol‐3‐yl)piperazin‐1‐yl)benzylidene)‐4‐methoxybenzohydrazide (**8c**).


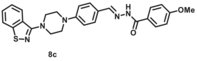
Yellow solid; yield 82%; mp: 178°C–180°C; ^1^H NMR (500 MHz, DMSO‐*d*_6_) *δ* 11.52 (s, 1H), 8.35 (s, 1H), 8.15 (s, 1H), 8.09 (d, *J* = 8.1 Hz, 1H), 7.90 (d, *J* = 8.8 Hz, 2H), 7.60 (dd, *J* = 15.1, 8.0 Hz, 3H), 7.48 (d, *J* = 7.4 Hz, 1H), 7.07 (dd, *J* = 14.0, 8.7 Hz, 4H), 3.84 (s, 3H), 3.62–3.60 (m, 4H), 3.50 (s, 4H); ^13^C NMR (125 MHz, DMSO‐*d*_6_) *δ* 163.82, 156.05, 152.56, 147.98, 143.92, 130.74, 130.25, 129.89, 128.73, 128.43, 127.79, 127.14, 125.15, 124.96, 124.84, 124.60, 121.59, 120.03, 117.96, 115.26, 114.14, 55.88, 49.87, 47.68; HRMS‐QTOF (ESI): *m/z* calcd. for [M + H]^+^ C26H26N5O2S 472.1802; observed 472.1792.

(*E*)‐N′‐(4‐(4‐(benzo[d]isothiazol‐3‐yl)piperazin‐1‐yl)benzylidene)‐4‐chlorobenzohydrazide (**8d**).


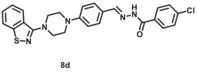
White solid; yield 76%; mp: 207°C–209°C; ^1^H NMR (500 MHz, DMSO‐*d*_6_) *δ* 11.70 (s, 1H), 8.35 (s, 1H), 8.15 (s, 1H), 8.09 (d, *J* = 8.1 Hz, 1H), 7.93 (d, *J* = 8.3 Hz, 2H), 7.63–7.59 (m, 5H), 7.47 (s, 1H), 7.09 (d, *J* = 8.6 Hz, 2H), 3.61 (d, *J* = 4.2 Hz, 4H), 3.51 (s, 4H).^13^C NMR (125 MHz, DMSO‐*d*_6_) *δ* 163.81, 162.19, 152.57, 148.93, 136.82, 132.91, 129.94, 128.99, 128.91, 128.44, 127.79, 124.96, 124.83, 124.60, 121.60, 115.21, 49.86, 47.61. HRMS‐QTOF (ESI): *m/z* calcd. for [M + H]^+^ C_25_H_23_ClN_5_OS 476.1306; observed 476.1308.

(*E*)‐N′‐(4‐(4‐(benzo[d]isothiazol‐3‐yl)piperazin‐1‐yl)benzylidene)‐4‐(trifluoromethyl)benzohydrazide (**8e**).


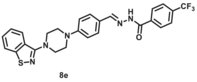
White solid; yield 69%; mp: 212°C–214°C; ^1^H NMR (500 MHz, DMSO‐*d*_6_) *δ* 11.84 (s, 1H), 8.38 (s, 1H), 8.15–8.09 (m, 4H), 7.91 (d, *J* = 8.2 Hz, 2H), 7.64 (d, *J* = 8.7 Hz, 2H), 7.59 (t, *J* = 7.5 Hz, 1H), 7.48 (t, *J* = 7.5 Hz, 1H), 7.09 (d, *J* = 8.6 Hz, 2H), 3.61 (d, *J* = 5.1 Hz, 4H), 3.52 (s, 4H); ^13^C NMR (125 MHz, DMSO‐*d*_6_) *δ* 167.62, 166.53, 163.82, 162.09, 152.55, 149.38, 148.63, 143.65, 142.24, 134.68, 129.86, 129.71, 129.00, 128.95, 128.45, 127.74, 127.15, 126.03, 125.89, 124.97, 124.60, 121.60, 115.18, 112.77, 49.86, 47.56. HRMS‐QTOF (ESI): *m/z* calcd. for [M + H]^+^ C_26_H_23_F_3_N_5_OS 510.1570; observed 510.1558.

(*E*)‐N′‐(4‐(4‐(benzo[d]isothiazol‐3‐yl)piperazin‐1‐yl)benzylidene)‐3‐chlorobenzohydrazide (**8f**).


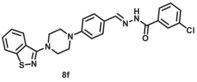
Yellow solid; yield 73%; mp: 188°C–190°C; ^1^H NMR (500 MHz, DMSO‐*d*_6_) *δ* 11.73 (s, 1H), 8.35 (s, 1H), 8.14 (d, *J* = 8.2 Hz, 1H), 8.09 (d, *J* = 8.1 Hz, 1H), 7.95 (s, 1H), 7.87 (d, *J* = 7.8 Hz, 1H), 7.76 (d, *J* = 8.9 Hz, 1H), 7.66 (d, *J* = 8.0 Hz, 1H), 7.63 (d, *J* = 8.8 Hz, 1H), 7.59–7.55 (m, 2H), 7.47 (d, *J* = 7.8 Hz, 1H), 7.14–7.08 (m, 2H), 3.63–3.61 (m, 4H), 3.52 (d, *J* = 5.0 Hz, 4H); ^13^C NMR (125 MHz, DMSO‐*d*_6_) *δ* 163.81, 161.81, 155.14, 152.56, 149.14, 136.18, 133.72, 131.98, 131.82, 130.93, 128.95, 128.43, 127.79, 127.72, 126.83, 124.96, 124.75, 124.60, 121.59, 115.19, 113.88, 49.86, 47.59HRMS‐QTOF (ESI): *m/z* calcd. for [M + H]^+^ C_25_H_23_ClN_5_OS 476.1306; observed 476.1309.

(*E*)‐N′‐(4‐(4‐(benzo[d]isothiazol‐3‐yl)piperazin‐1‐yl)benzylidene)‐4‐bromobenzohydrazide (**8 g**).


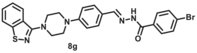
Brown solid; yield 79%; mp: 218°C–220°C; ^1^H NMR (500 MHz, DMSO‐*d*_6_) *δ* 11.68 (s, 1H), 8.35 (s, 1H), 8.15 (s, 1H), 8.09 (d, *J* = 8.1 Hz, 1H), 7.86 (d, *J* = 8.5 Hz, 2H), 7.74 (d, *J* = 8.5 Hz, 2H), 7.62 (s, 1H), 7.58 (d, *J* = 7.9 Hz, 1H), 7.47 (t, *J* = 7.6 Hz, 2H), 7.09 (d, *J* = 8.8 Hz, 2H), 3.62 (s, 4H), 3.52 (d, *J* = 5.0 Hz, 4H); ^13^C NMR (125 MHz, DMSO‐*d*_6_) *δ*163.80, 161.46, 152.56, 148.42, 144.76, 139.06, 135.17, 134.93, 128.93, 128.43, 127.79, 127.01, 124.96, 124.60, 121.59, 115.33, 49.85, 47.61. HRMS‐QTOF (ESI): *m/z* calcd. for [M + H]^+^ C_25_H_23_BrN_5_OS 522.0801; observed 522.0777.

(*E*)‐N′‐(4‐(4‐(benzo[d]isothiazol‐3‐yl)piperazin‐1‐yl)benzylidene)‐4‐phenoxybenzohydrazide (**8 h**).


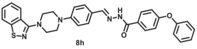
Yellow solid; yield 87%; mp: 213°C–215°C; ^1^H NMR (500 MHz, DMSO‐*d*_6_) *δ* 11.60 (s, 1H), 8.35 (s, 1H), 7.62 (d, *J* = 8.6 Hz, 2H), 7.58 (d, *J* = 7.3 Hz, 1H), 7.48 (d, *J* = 7.5 Hz, 2H), 7.46 (s, 2H), 7.22 (d, *J* = 7.4 Hz, 1H), 7.08 (s, 1H), 3.61 (d, *J* = 5.1 Hz, 4H), 3.51 (s, 4H); ^13^C NMR (125 MHz, DMSO‐*d*_6_) *δ* 163.82, 156.05, 152.56, 148.34, 138.39, 130.75, 130.25, 128.80, 128.44, 127.79, 124.97, 124.84, 124.60, 121.60, 120.03, 117.96, 115.24, 78.10, 49.87, 47.65.HRMS‐QTOF (ESI): *m/z* calcd. for [M + H]^+^ C_31_H_28_N_5_O_2_S 534.1958; observed 534.1945.

(*E*)‐N′‐(4‐(4‐(benzo[d]isothiazol‐3‐yl)piperazin‐1‐yl)benzylidene)cyclopropanecarbohydrazide (**8i**).


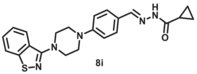
White solid; yield 70%; mp: 171°C–173°C; ^1^H NMR (500 MHz, DMSO‐*d*_6_) *δ* 11.27 (d, *J* = 119.7 Hz, 1H), 8.15–7.93 (m, 3H), 7.54 (dd, *J* = 32.7, 24.6 Hz, 4H), 7.05 (s, 2H), 3.60 (s, 4H), 3.48 (s, 4H), 1.62 (s, 1H), 0.85 (s, 2H), 0.77 (s, 2H); ^13^C NMR (125 MHz, DMSO‐*d*_6_) *δ* 174.65, 169.34, 163.82, 152.56, 146.15, 143.61, 128.63, 128.43, 128.32, 127.80, 124.96, 124.59, 121.59, 115.35, 115.25, 49.87, 47.78, 47.71, 13.30, 10.20, 8.14, 7.16. HRMS‐QTOF (ESI): *m/z* calcd. for [M + H]^+^ C_22_H_24_N_5_OS 406.1696; observed 406.1685.

(*E*)‐N′‐(4‐(4‐(benzo[d]isothiazol‐3‐yl)piperazin‐1‐yl)benzylidene)‐3‐hydroxy‐1‐naphthohydrazide (**8j**).


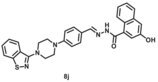
Yellow solid; yield 78%; mp: 198°C–200°C; ^1^H NMR (500 MHz, DMSO‐*d*_6_) *δ* 11.89 (s, 1H), 11.62 (s, 1H), 8.48 (s, 1H), 8.37 (s, 1H), 8.14 (d, *J* = 8.2 Hz, 1H), 8.09 (d, *J* = 8.1 Hz, 1H), 7.91 (d, *J* = 8.1 Hz, 1H), 7.76 (d, *J* = 8.4 Hz, 1H), 7.66 (d, *J* = 8.7 Hz, 2H), 7.59 (t, *J* = 7.5 Hz, 1H), 7.53–7.46 (m, 2H), 7.36 (t, *J* = 7.4 Hz, 1H), 7.32 (s, 1H), 7.11 (d, *J* = 8.8 Hz, 2H), 3.62 (d, *J* = 5.3 Hz, 4H), 3.53 (d, *J* = 4.9 Hz, 4H); ^13^C NMR (125 MHz, DMSO‐*d*_6_) *δ*163.81, 154.98, 152.66, 152.57, 149.47, 136.34, 130.46, 129.08, 128.65, 128.44, 127.79, 127.21, 126.30, 124.97, 124.60, 124.21, 121.60, 120.46, 115.17, 111.09, 49.86, 47.57.HRMS‐QTOF (ESI): *m/z* calcd. for [M + H]^+^ C_29_H_26_N_5_O_2_S 508.1802; observed 508.1786.

(*E*)‐N′‐(4‐(4‐(benzo[d]isothiazol‐3‐yl)piperazin‐1‐yl)benzylidene)isonicotinohydrazide (**8k**).


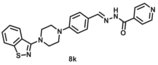
Yellow solid; yield 81%; mp: 223°C–225°C; ^1^H NMR (500 MHz, DMSO‐*d*_6_) *δ* 11.87 (s, 1H), 8.79 (d, *J* = 5.8 Hz, 2H), 8.37 (s, 1H), 8.14 (d, *J* = 8.2 Hz, 1H), 8.09 (d, *J* = 8.1 Hz, 1H), 7.84 (dd, *J* = 4.5, 1.5 Hz, 2H), 7.64 (d, *J* = 8.8 Hz, 2H), 7.59 (dd, *J* = 11.1, 4.0 Hz, 1H), 7.49–7.46 (m, 1H), 7.09 (d, *J* = 8.9 Hz, 2H), 3.63–3.60 (m, 4H), 3.53 (d, *J* = 5.3 Hz, 4H); ^13^C NMR (125 MHz, DMSO‐*d*_6_) *δ* 163.82, 161.95, 157.50, 152.57, 148.59, 131.99, 131.51, 128.89, 128.85, 128.43, 128.00, 124.97, 124.60, 121.59, 115.23, 49.87, 47.64.HRMS‐QTOF (ESI): *m/z* calcd. for [M + H]^+^ C_24_H_23_N_6_OS 443.1649; observed 443.1630.

(*E*)‐N′‐(4‐(4‐(benzo[d]isothiazol‐3‐yl)piperazin‐1‐yl)benzylidene)nicotinohydrazide (**8l**).


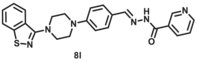
White solid; yield 57%; mp: 218°C–220°C; ^1^H NMR (500 MHz, DMSO‐*d*_6_) *δ* 11.81 (s, 1H), 9.06 (s, 1H), 8.75 (s, 1H), 8.35 (s, 1H), 8.25 (d, *J* = 7.1 Hz, 1H), 8.14 (d, *J* = 7.9 Hz, 1H), 8.09 (d, *J* = 8.1 Hz, 1H), 7.63 (s, 1H), 7.58 (d, *J* = 7.2 Hz, 2H), 7.48 (t, *J* = 7.2 Hz, 2H), 7.09 (d, *J* = 8.1 Hz, 2H), 3.62 (s, 4H), 3.52 (s, 4H).; ^13^C NMR (125 MHz, DMSO‐*d*_6_) *δ* 164.13, 161.59, 157.49, 149.47, 145.78, 136.34, 130.46, 129.08, 128.65, 128.44, 127.79, 127.21, 126.30, 124.97, 124.60, 124.21, 121.60, 120.46, 115.17, 111.09, 49.86, 47.57.HRMS‐QTOF (ESI): *m/z* calcd. for [M + H]^+^ C_24_H_23_N_6_OS 443.1649; observed 443.1630.

(*E*)‐N′‐(4‐(4‐(benzo[d]isothiazol‐3‐yl)piperazin‐1‐yl)benzylidene)thiophene‐2‐carbohydrazide (**8m**).


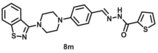
Yellow solid; yield 72%; mp: 193°C–195°C; ^1^H NMR (500 MHz, DMSO‐*d*_6_) *δ* 12.07 (s, 1H), 9.26 (s, 1H), 8.92 (d, *J* = 1.6 Hz, 1H), 8.78 (s, 1H), 8.54 (s, 1H), 8.16–8.08 (m, 2H), 7.64–7.59 (m, 3H), 7.48 (t, *J* = 7.5 Hz, 1H), 7.09 (d, *J* = 8.5 Hz, 2H), 3.62 (s, 4H), 3.52 (s, 4H); ^13^C NMR (125 MHz, DMSO‐*d*_6_) *δ* 167.43, 163.80, 159.72, 159.55, 155.35, 152.69, 152.56, 150.57, 148.50, 144.48, 144.36, 143.92, 143.73, 142.03, 129.89, 129.09, 128.43, 127.14, 124.96, 124.59, 115.16, 49.84, 47.56.HRMS‐QTOF (ESI): *m/z* calcd. for [M + H]^+^ C_23_H_21_N_5_OS_2_ 448.1260; observed 448.1239.

(*E*)‐N′‐(4‐(4‐(benzo[d]isothiazol‐3‐yl)piperazin‐1‐yl)benzylidene)pyrazine‐2‐carbohydrazide (**8n**).


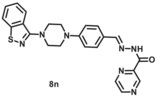
White to pale yellow solid; yield 80%; mp: 236°C–238°C; ^1^H NMR (500 MHz, DMSO‐*d*_6_) *δ* 11.71 (s, 1H), 8.35 (s, 1H), 8.15 (s, 1H), 8.09 (d, *J* = 8.1 Hz, 1H), 7.86 (d, *J* = 8.5 Hz, 2H), 7.74 (d, *J* = 8.5 Hz, 2H), 7.62 (s, 1H), 7.58 (d, *J* = 7.9 Hz, 1H), 7.47 (d, *J* = 7.7 Hz, 1H), 7.09 (d, *J* = 8.8 Hz, 2H), 3.62 (s, 4H), 3.52 (d, *J* = 5.0 Hz, 4H); ^13^C NMR (125 MHz, DMSO‐*d*_6_) *δ* 163.81, 162.30, 152.57, 148.95, 133.26, 131.93, 130.12, 128.91, 128.44, 127.79, 125.74, 124.96, 124.83, 124.60, 121.59, 115.20, 49.86, 47.61. HRMS‐QTOF (ESI): *m/z* calcd. for [M + H]^+^ C_23_H_22_N_7_OS 444.1601; observed 444.1608.

### 5.2 Antimycobacterial Activity

#### 5.2.1 Strains and Growing Conditions

The attenuated *Mycobacterium tuberculosis* H_37_Rv mc^2^6230 strain was obtained from BEI Resources, NIAID, NIH (Manassas, VA, USA). *MTB* H_37_Rv MC^2^ 6230 was used to evaluate the compounds in vitro to determine MIC values. The bacteria were cultured in Middlebrook 7H9 liquid broth medium supplemented with D‐pantothenic acid (24 mg/L), L‐leucine (50 mg/L), L‐methionine (50 mg/L), L‐arginine (200 mg/L), 0.2% glycerol, 0.05% Tween 80, and 10% Albumin‐Dextrose‐Saline (ADS) supplement at 37°C with 5% CO_2_. Isoniazid, a standard first‐line drug, was used as a positive control in the biological assays.

#### 5.2.2 Method

The compound's MIC was determined in vitro using the liquid broth micro‐dilution method, as described previously (Wiegand et al. 2008). Mycobacterial suspensions were cultured in 7H9 liquid broth medium supplemented with D‐pantothenic acid (24 mg/L), L‐leucine (50 mg/L), L‐methionine (50 mg/L), L‐arginine (200 mg/L), 0.2% glycerol, 0.05% Tween 80, and 10% Albumin‐Dextrose‐Saline (ADS). The suspensions, in the log phase (OD600 range of 0.4–0.6), equivalent to 1.0 × 108 colony‐forming units (CFU)/mL, were diluted to an OD600 of 0.005 (1.0 × 106 CFU/mL). The test compounds were serially diluted twice in 100 μL of 7H9 liquid broth medium in each well of the microtiter plate. Subsequently, 100 μL of *Mtb* H_37_Rv MC^2^ 6230 culture (OD600 0.005) was added to each well. The assay plates were then incubated at 37°C for 10 days in a 5% CO_2_ environment. Following the incubation period, the plates were visually inspected, and the MIC was determined as the lowest concentration of the compound that showed no turbidity. *Mycobacterium tuberculosis* H_37_Rv (ATCC 27294) was obtained from the American Type Culture Collection (ATCC, Manassas, VA, USA). Cytotoxicity studies were performed using the THP‐1 human monocytic cell line, obtained from the American Type Culture Collection (ATCC, Cat. No. TIB‐202, Manassas, VA, USA).

#### 5.2.3 Method

##### 5.2.3.1 In Silico Studies

The crystal structures of InhA (PDB 2X23) (Luckner et al. 2010; Prasad et al. 2021), were downloaded from the Protein Data Bank (RCSB.org). All used structures were prepared using the Protein Preparation module implemented in the Maestro Schrödinger suite, assigning bond orders, adding hydrogens, deleting water molecules, and optimizing H‐bonding networks. Finally, energy minimization with a Root Mean Squared Deviation (RMSD) value of 0.30 was applied using an Optimized Potential for Liquid Simulation (OPLS4) force field. The 3D ligand structures were prepared by Maestro and evaluated for their ionization states at pH 7.3 ± 1.0 with Epik. The conjugate gradient method in Macromodel was used for energy minimization (maximum iteration number: 2500; convergence criterion: 0.05 Kcal/mol/Å2). Grids for docking were centered at the centroid of the complexed ligand.

## Author Contributions

**Vivek Sharma:** conceptualization, investigation, funding acquisition, writing – original draft, methodology, validation, visualization, writing – review and editing, software, formal analysis, data curation. **Arun Sharma:** supervision, writing – review and editing, resources. **Ruchi Poria:** supervision, project administration, resources. **Pardeep Kumar:** writing – review and editing, supervision.

## Funding

The authors have nothing to report.

## Conflicts of Interest

The authors declare no conflicts of interest.

## Supporting information


**Table S1:** ADME properties of all the synthesized molecules.

## Data Availability

The data that supports the findings of this study are available in Appendix S1 of this article.
