# Supplementary material for: Design, Synthesis, Antimycobacterial Evaluation, and In Silico Investigation of Benzisothiazole‐Based Hydrazide Derivatives as Potential Anti‐Tuberculosis Agents
Source: Chem Biol Drug Des. 2026 Sep 19;108(3):e70405. doi: 10.1111/cbdd.70405 (PMC13589221; doi:10.1111/cbdd.70405)
Supplement: Supplementary file 1 [file CBDD-108-e70405-s001.docx]

**Supporting information**

**Design, Synthesis, Antimycobacterial Evaluation, and *In Silico* Investigation of Benzisothiazole-Based Hydrazide Derivatives as Potential Anti-Tuberculosis Agents**

Vivek Sharma^1^, Arun Sharma^1^, Ruchi Poria^2^, Pardeep Kumar^2*^

^1^ Department of Chemistry U.G.C. Centre of Advanced Studies Guru Nanak Dev University Amritsar, 143005, India

^2^ Swami dayanand post graduate institute of pharmaceutical sciences rohtak, Pandit Bhagwat Dayal Sharma University of Health and Sciences 124001 Rohtak, Haryana, India

*Correspondence:

Dr. Pardeep Kumar

Swami dayanand post graduate institute of pharmaceutical sciences rohtak, Pandit Bhagwat Dayal Sharma University of Health and Sciences, Rohtak, Haryana, India 124001.

Email: [kumarpardeep061995@gmail.com](mailto:kumarpardeep061995@gmail.com)

**Table S-1 ADME properties of all the synthesized molecules**

| **Test Compounds** | **MW** | **HB Donors** | **HB Accptors** | **Num. rotatable bonds** | **TPSA** |
| --- | --- | --- | --- | --- | --- |
| **8a** | **441.55** | **1** | **3** | **6** | **89.07 Å²** |
| **8b** | **497.65** | **1** | **3** | **7** | **89.07 Å²** |
| **8c** | **471.57** | **1** | **4** | **7** | **98.30 Å²** |
| **8d** | **475.99** | **1** | **3** | **6** | **89.07 Å²** |
| **8e** | **509.55** | **1** | **6** | **7** | **89.07Å²** |
| **8f** | **475.99** | **1** | **3** | **6** | **89.07 Å²** |
| **8g** | **520.44** | **1** | **3** | **6** | **89.07 Å²** |
| **8h** | **533.64** | **1** | **4** | **8** | **98.30 Å²** |
| **8i** | **405.52** | **1** | **3** | **6** | **89.07 Å²** |
| **8j** | **507.61** | **2** | **4** | **6** | **109.30 Å²** |
| **8k** | 442.54 | **1** | **4** | **6** | **101.96 Å²** |
| **8l** | **442.54** | **1** | **4** | **6** | **101.96 Å²** |
| **8m** | **447.58** | **1** | **3** | **6** | **117.31 Å²** |
| **8n** | **443.52** | **1** | **5** | **6** | **114.85 Å²** |
| **Isoniazid** | **137.14** | **3.00** | **4.50** | **2** | **68.01 Å²** |

^1^H NMR (500 MHz, DMSO-*d_6_*) spectrum of compound **8a**

^13^C NMR (125 MHz, DMSO-*d_6_*) spectrum of compound **8a**

Mass spectrum of compound **8a**^1^H NMR (500 MHz, DMSO-*d_6_*) spectrum of compound **8b**^13^C NMR (125 MHz, DMSO-*d_6_*) spectrum of compound **8b**

HRMS-QTOF (ESI): *m/z* calcd. for [M+H]^+^ C_25_H_24_N_5_OS 442.1696; observed 442.1697

Mass spectrum of compound **8b**

HRMS-QTOF (ESI): *m/z* calcd. for [M+H]^+^ C_29_H_32_N_5_OS 498.2322; observed 498.2288

^1^H NMR (500 MHz, DMSO-*d_6_*) spectrum of compound **8c**^13^C NMR (125 MHz, DMSO-*d_6_*) spectrum of compound **8c**

Mass spectrum of compound **8c**^1^H NMR (500 MHz, DMSO-*d_6_*) spectrum of compound **8d**^13^C NMR (125 MHz, DMSO-*d_6_*) spectrum of compound **8d**

HRMS-QTOF (ESI): *m/z* calcd. for [M+H]^+^ C_26_H_26_N_5_O_2_S 472.1802; observed 472.1792

Mass spectrum of compound **8d**

HRMS-QTOF (ESI): *m/z* calcd. for [M+H]^+^ C_25_H_23_ClN_5_OS 476.1306; observed 476.1308

^1^H NMR (500 MHz, DMSO-*d_6_*) spectrum of compound **8e**^13^C NMR (125 MHz, DMSO-*d_6_*) spectrum of compound **8e**

Mass spectrum of compound **8e**

HRMS-QTOF (ESI): *m/z* calcd. for [M+H]^+^ C_26_H_23_F_3_N_5_OS 510.1570; observed 510.1558.

^1^H NMR (500 MHz, DMSO-*d_6_*) spectrum of compound **8f**

^13^C NMR (125 MHz, DMSO-*d_6_*) spectrum of compound **8f**

Mass spectrum of compound **8f**

HRMS-QTOF (ESI): *m/z* calcd. for [M+H]^+^ C_25_H_23_ClN_5_OS 476.1306; ; observed 476.1309

^1^H NMR (500 MHz, DMSO-*d_6_*) spectrum of compound **8g**^13^C NMR (125 MHz, DMSO-*d_6_*) spectrum of compound **8g**

Mass spectrum of compound **8g**

HRMS-QTOF (ESI): *m/z* calcd. for [M+H]^+^ C_25_H_23_BrN_5_OS 522.0801; observed 522.0777

^1^H NMR (500 MHz, DMSO-*d_6_*) spectrum of compound **8h**

^13^C NMR (125 MHz, DMSO-*d_6_*) spectrum of compound **8h**

Mass spectrum of compound **8h**^1^H NMR (500 MHz, DMSO-*d_6_*) spectrum of compound **8i**

HRMS-QTOF (ESI): *m/z* calcd. for [M+H]^+^ C_31_H_28_N_5_O_2_S 534.1958; observed 534.1945

^13^C NMR (125 MHz, DMSO-*d_6_*) spectrum of compound **8i**

Mass spectrum of compound **8i**^1^H NMR (500 MHz, DMSO-*d_6_*) spectrum of compound **8j**^13^C NMR (125 MHz, DMSO-*d_6_*) spectrum of compound **8j**

HRMS-QTOF (ESI): *m/z* calcd. for [M+H]^+^ C_22_H_24_N_5_OS 406.1696; observed 406.1685

Mass spectrum of compound **8j**

HRMS-QTOF (ESI): *m/z* calcd. for [M+H]^+^ C_29_H_26_N_5_O_2_S 508.1802; observed 508.1786

^1^H NMR (500 MHz, DMSO-*d_6_*) spectrum of compound **8k**^13^C NMR (125 MHz, DMSO-*d_6_*) spectrum of compound **8k**Mass spectrum of compound **8k**^1^H NMR (500 MHz, DMSO-*d_6_*) spectrum of compound **8l**^13^C NMR (125 MHz, DMSO-*d_6_*) spectrum of compound **8l**

HRMS-QTOF (ESI): *m/z* calcd. for [M+H]^+^ C_24_H_23_N_6_OS 443.1649; observed 443.1630

Mass spectrum of compound **8l**

HRMS-QTOF (ESI): *m/z* calcd. for [M+H]^+^ C_24_H_23_N_6_OS 443.1649; observed 443.1630

^1^H NMR (500 MHz, DMSO-*d_6_*) spectrum of compound **8m**^13^C NMR (125 MHz, DMSO-*d_6_*) spectrum of compound **8m**

Mass spectrum of compound **8m**^1^H NMR (500 MHz, DMSO-*d_6_*) spectrum of compound **8n**

HRMS-QTOF (ESI): *m/z* calcd. for [M+H]^+^ C_23_H_21_N_5_OS_2_ 448.1260; observed 448.1239

^13^C NMR (125 MHz, DMSO-*d_6_*) spectrum of compound **8n**Mass spectrum of compound **8n**

HRMS-QTOF (ESI): *m/z* calcd. for [M+H]^+^ C_23_H_22_N_7_OS 444.1601; observed 444.1608
